# The role of human milk feeds on inotrope use in newborn infants with sepsis

**DOI:** 10.3389/fped.2023.1172799

**Published:** 2023-04-17

**Authors:** Elisenda Moliner-Calderón, Sergio Verd, Alfonso Leiva, Gemma Ginovart, Pia Moll-McCarthy, Josep Figueras-Aloy

**Affiliations:** ^1^ Neonatal Unit, Department of Paediatrics, Santa Creu I Sant Pau Hospital, Barcelona, Spain; ^2^ Pediatric Unit, La Vileta Surgery, Department of Primary Care, Palma de Mallorca, Spain; ^3^ Group of Cell Therapy and Tissue Engineering, Institut d'Investigació Sanitària Illes Balears (IdISBa), Palma de Mallorca, Spain; ^4^ Research Unit, Department of Primary Care, Palma de Mallorca, Spain; ^5^ Neonatal Unit, Department of Paediatrics, Germans Trias I Pujol Hospital, Badalona, Spain; ^6^ A & E Department, Manacor District Hospital, Manacor, Spain; ^7^ Neonatal Unit, ICGON, Clinic Hospital, Barcelona, Spain

**Keywords:** neonate, premature infant, breast-feeding, human milk, sepsis, hypotension, oxidative stress, dopamine

## Abstract

**Background:**

Regarding neonatal hypotension, there is no certainty as to whether inotrope properties are beneficial or whether they may be harmful. However, given that the antioxidant content of human milk plays a compensatory role in neonatal sepsis and that human milk feeding has direct effects in modulating the cardiovascular function of sick neonates, this research hypothesized that human milk feeds might predict lower requirements of vasopressors in the management of neonatal septic shock.

**Method:**

Between January 2002 and December 2017, all late preterm and full-term infants attending a neonatal intensive care unit, with clinical and laboratory findings of bacterial or viral sepsis, were identified in a retrospective study. During their first month of life, data on feeding type and early clinical characteristics were collected. A multivariable logistic regression model was constructed to determine the impact of human milk on the use of vasoactive drugs in septic newborns.

**Results:**

322 newborn infants were eligible to participate in this analysis. Exclusively formula-fed infants were more likely to be delivered *via* C-section, to have a lower birth weight and a lower 1-minute Apgar score than their counterparts. Human milk-fed newborns had 77% (adjusted OR = 0.231; 95% CI: 0.07–0.75) lower odds of receiving vasopressors than exclusively formula-fed newborns.

**Conclusion:**

We report that any human milk feeding is associated with a decrease in the need for vasoactive medications in sepsis-affected newborns. This observation encourages us to undertake further research to determine whether human milk feeds mitigate the use of vasopressors in neonates with sepsis.

## Introduction

Recent research (1) that aimed to reduce the use of inotropes resulted in a reduction of brain morbidities in premature babies; these findings add to serious efforts to clarify whether cerebral perfusion is improved or reduced with any inotrope use. Unfortunately, despite decades of use, we still have little evidence regarding the outcome of the various inotropes accepted for treating neonatal hypotension. A limited number of observational studies have addressed the long-term effects of vasoactive drugs. However, recruiting sick neonates for randomized controlled trials when they are unstable and require prompt intervention is extremely challenging (2). Given this, it is wise of us to try and find more ways to narrow the use of agents that may be of no benefit or potentially have adverse consequences.

Although neonates generate a relatively lower amount of inflammatory cytokines than adults, neonates with septic shock have dysregulated cytokine production, which is linked to illness severity (3). Inflammatory pathways activated in septic shock directly impact the vascular tone and cardiac function. The release of inflammatory mediators can compromise endothelial wall integrity; subsequent endothelial leakage lowers circulating blood volume and, subsequently, cardiac preload (4, 5). Bacteremic neonates have been found to have higher levels of nitric oxide and endothelin compared to their non-infected counterparts. High levels of endothelin have been correlated with myocardial dysfunction in sepsis (6).

Remarkably, systemic inflammatory response and additional compromise to perfusion occur when anti-inflammatory mechanisms do not adequately counteract pro-inflammatory pathways. Antioxidant and anti-inflammatory defenses mature during late gestation (7). Thus, preterm neonates have diminished anti-inflammatory mechanisms at birth. On the other hand, the process leading to septic shock is maintained by a cascade of inflammatory mechanisms and simultaneous increased production of free radicals. Since human milk (HM) possesses a much more potent antioxidant potential than infant formula (8), it appears to serve as the primary antioxidant source for preterm or sick neonates. Although we have not found previous research on the link between HM feeding and an overall decline in the use of vasoactive medications, the antioxidant role of HM and the critical role that oxidative stress plays in worsening the hemodynamic presentation of neonatal sepsis generate the hypothesis that HM feeding might predict lower requirements of vasopressors in the management of neonatal septic shock.

In this regard, we have analyzed the link between HM feeding and the use of vasopressors in a long-term cohort of newborn infants with sepsis admitted to our neonatal unit.

## Methods

### Design

This is a secondary analysis of data collected for a doctoral thesis intended to explore the clinical and laboratory characteristics of neonates with viral or bacterial sepsis (9). Previous research contains complete details of the methodological processes (10). In brief, this study was carried out using a retrospective review of electronic medical records of late preterm and full-term newborn infants.

### Study population and period

Between January 2002 and December 2017, all late preterm and full-term infants up to 28 days of age with clinical and laboratory findings of bacterial or viral sepsis were identified in a cohort study. All of them attended the neonatal intensive care unit (NICU) of Sant Pau Hospital, a tertiary referral unit in Barcelona, Spain, with approximately 350 admissions per year and a bed occupancy rate of 90%; the unit consists of 10 intensive care and 7 high-care beds.

### Data collection

The NICU's computerized system provided retrospective data on maternal parity and gravida, maternal diseases, infant demographic and perinatal characteristics, feeding type, and early clinical features. In addition, data on any HM or exclusive formula feeding (EF) were collected during the first month of life. All HM was mother's own milk; donor's milk is reserved for very premature infants. Fresh or frozen mother's own milk was used. These infants were not admitted to the NICU from home but from the Maternity unit. We chose to focus on the first month of life because it significantly impacts short-term outcomes. For exploratory analyses, we examined the use of antihypotensive treatments until hospital discharge.

The primary outcome of this study was to examine the use of vasopressors in newborn infants with a history of sepsis, intending to evaluate if exclusive formula feeding predicts vasopressor support practices. In this preliminary analysis, infant feeding type was an independent variable, and vasopressor support was a dependent variable.

Secondary outcomes included the following clinical characteristics based on feeding type. Neonatal status at birth, metabolic and/or respiratory acidosis, hypotension, abnormal neurological examination, abnormal brain scan, meningitis, positive blood cultures, blood cells count, type of respiratory support, discharge weight, and days spent in the hospital. The clinical information from birth until Hospital discharge has been gathered in a database, but a detail of the timing of each clinical characteristic was not available.

### Statistical analysis

We used descriptive statistics to generate counts and percentages for the mode of feeding among newborn infants in the registry. Fisher's Exact or *t*-tests were used to compare infant and maternal characteristics or outcomes; *p*-values were obtained from bivariate comparisons as a function of each individual risk factor. A multivariable logistic regression model was constructed to determine the impact of milk type on the primary outcome and the independent contribution of each factor to neonatal outcomes. This model included variables associated with exposure or variables highly predictive of the primary outcomes. Backward selection was used to remove these covariables from the multivariable logistic regression model if they no longer had significance when added to the model. Because of collinearity, heart failure was not included in the adjusted model. Outcomes were compared between the HM and EF groups with odds ratios calculated with logistic regression fit by generalized estimating equations to account for paired data. There were no methods used to account for any potential bias. The SPSS software version 21.0 (SPSS Chicago, IL, United States) was used for statistical analysis and data management.

### Ethical statement

The Santa Creu & Sant Pau University Hospital's Clinical Studies Ethics Committee approved this study on December 20, 2020, with decision number: IIBSP-ENT-2020-152.

Because the study was conducted retrospectively over a two-decade period, and the data was scanned from patient files, no informed consent was obtained.

## Results

During the study period (2002–2017), 4,210 neonates were admitted to the participating NICU, 7.6% (*n* = 322; 260 in HM group and 62 in EF group) of whom met inclusion criteria and were eligible to participate in the final analysis. Similar to what was published in an earlier study of these cohorts examining the relationship between type of feeding and type of respiratory support (10), baseline characteristics of neonates who received any amount of HM and neonates who received only formula are summarized in Table 1. The mean age of newborns on admission to the neonatal unit was 4.3 days in cases of bacterial sepsis and 12.9 days in cases of viral sepsis, and 20% of infants with bacterial sepsis required vasoactive support compared to 2% of infants with viral sepsis. EF infants were more likely to be delivered *via* C-section, had a lower birth weight, and had a lower 1 min Apgar score than HM infants. However, these relationships did not persist when introduced in the multivariate model. We found no other background differences in bivariate analysis.

**Table 1:** Patents’ characterstics.

	Any human milk feeding, *n* = 260	Exclusive formula feeding, *n* = 62	*p* value
Gestational hypertension	21 (8.07%)	6 (9.7%)	0.607
Gestational diabetes	8 (3.0)	2 (3.3)	1.000
Chrioamnionitis	63 (24.0)	20 (32.2)	0.218
Group B strep positive mother	36 (13.8)	5 (8.3)	0.512
Gestational age, weeks	38.59 (2.08)	38.01 (2.27)	0.056
Delivery type: eutocic/instrumental/C-section	147/43/72	29/3/28	0.0045**
Delivery type: vaginal/C-section	190/72	32/28	0.0052**
Singletons/twins	249/13	53/7	0.071
Apgar 1 min	8 (2)	7 (3)	0.036*
Apgar 5 min	9 (1)	9 (2)	1.000
Birth weight, g	3,116 (639)	2,893 (637)	0.010
Height, cm	48.6 (2.84)	48.22 (2.69)	0.322
Cranial circumference, cm	33.83 (2.02)	33.37 (1.94)	0.093
Boys/Girls	136/126	34/26	0.567

Data expressed as number (%) or mean (standard deviation). Fisher's exact test or *t*-test comparison between neonates with any human milk feeding and exclusive formula feeding.

* *p* < 0.05.

** *p* < 0.01.

Table 2 compares short-term neonatal outcomes by feeding method. On bivariate analysis, there was a higher proportion of hipotonia among EF infants than among HM infants (54% vs. 23%, *p* = 0.045); and the proportion of respiratory distress was higher among EF infants than among HM infants (56% vs. 39%, *p* = 0.020). As a result, the same applied to the proportion of infants needing some form of respiratory support (43% vs. 27%, *p* = 0.019). On the other hand, the neutrophil blood count was higher among EF infants than among HM infants (10,358/µl vs. 7,623/µl, *p* = 0.005). Interestingly, 22% of EF infants had confirmed viral or bacterial meningitis vs. 42% of HM infants (*p* = 0.038). Finally, 8.7% of HM neonates required vasopressor support, compared to 25% of EF neonates (OR = 0.28; 95% CI: 0.14–0.59; *p* < 0.001). Other short-term neonatal outcomes did not differ between the two groups.

**Table 2:** Clinical or laboratory outcomes by feeding type.

	Any human milk feeding, *n* = 260	Exclusive formula feeding, *n* = 62	*p* value
Apnea	47 (18.9)	17 (28.8)	0.074
Abnormal alertness state	143 (54.5)	27 (45)	0.198
Hypotonia	59 (22.5)	21 (53.8)	0.045*
Convulsions	10 (3.8)	4 (6.6)	0.304
Hypotension	43 (16.4)	16 (26.6)	0.094
Heart failure	7 (2.7)	4 (6.6)	0.128
Hepatitis	20 (7.6)	9 (15)	0.081
Fever	122 (46.5)	23 (38.3)	0.254
Rash	29 (11.0)	2 (3.3)	0.087
Vomiting	27 (10.3)	9 (15)	0.361
Neonatal acidosis	77 (29.7)	24 (40)	0.121
Positive blood culture	81 (30.9)	18 (30)	1.000
Bacterial/viral meningitis	84/113 (42.6)	7 (22.5)	0.038*
Leukocyte blood count/µl	15,317 (30,538)	23,784 (38,184)	0.103
Neutrophil blood count/µl	7,623 (5,805)	10,358 (6,980)	0.005**
Lymphocyte blood count/µl	3,896 (2,296)	4,384 (2,505)	0.197
Abnormal brain ultrasound	13 (5.9)	1 (1.9)	0.482
Pulmonary edema	13 (4.9)	7 (22.5)	0.071
Respiratory distress	103 (39.7)	34 (56.6)	0.020*
Any respiratory support	72 (27.4)	26 (43.3)	0.019*
Administered inotropes	23 (8.7%)	15/45 (25)	0.001***
Administered steroids	6 (2.2)	1 (1.9)	1.000
Administered antibiotics	212 (80.9)	53 (88.3)	0.194
Hospitalization, days	10 (9)	12 (9)	0.121
Discharge weight, g	3,425 (689)	3,256 (639)	0.083

Data expressed as number (%) or mean (standard deviation). Fisher's exact test or *t*-test comparison between neonates with any human milk feeding and exclusive formula feeding.

* *p* < 0.05.

** *p* < 0.01.

*** *p* < 0.001.

Logistic regression models were developed to examine the association between the use of vasopressors and feeding type. Meningitis, lower muscle tone, C-section, or postnatal steroids treatment were covariates that remained significant in the final model (Table 3). However, even when controlling for these covariates, HM newborns had 77% (adjusted OR = 0.231; 95% CI: 0.07–0.75) lower odds of receiving vasopressors than EF newborns.

**Table 3:** Multivariate logistic regression analysis of factors associated with higher vasopressor support.

Variable	Odds ratio	Standard error	*z*	*p *>* *|*z*|	95% CI
Human milk feeding	0.231	0.139	−2.43	0.015	0.071–0.752
Meningitis	0.242	0.127	−2.69	0.007	0.086–0.680
Lower muscle tone	3.239	2.276	2.43	0.015	1.308–12.203
C-section	3.426	2.080	2.03	0.043	1.042–11.264
Postnatal steroids	96.757	206.68	2.15	0.031	1.500–6239.6

CI, confidence interval; *P*>|*z*|, Corresponding Probability for the Reduced Logistic Model; *z*, Estimated *Z*-Score.

## Discussion

We found that any HM is linked to much better cardiovascular outcomes than EF. Data on exclusive HM feeding are not available in this study. However, the protecting role of any HM is supported by systematic reviews, which show that a minimum intake of 7 ml/kg/day of HM during the first 42 days is linked to a lower incidence of bronchopulmonary dyspasia and that there is conflicting evidence regarding the impact of high vs. low doses of HM on both this condition or retinopathy of prematurity (11).

This retrospective study at the level of a single NICU has explored the link between early feeding type and the administration of vasopressors. Following our initial hypothesis, after adjusting for confounding by indication, infants in the EF groups received vasopressor treatment more frequently than the HM group. As far as we know, this is the pioneer descriptive study in this field. In addition, this analysis included a large number of newborn infants and used multivariable statistical methods, which contributed to the findings' external validity.

We also found a positive association between any HM feeding and the rate of meningitis, a negative association between any HM feeding and hypotonia, and neutrophil counts were significantly higher in EF neonates than in HM neonates. We do not have an explanation for our results on meningitis. We also found a positive association between any HM feeding and the rate of meningitis, a negative association between any HM feeding and hypotonia, and neutrophil counts were significantly higher in EF neonates than in HM neonates. We do not have an explanation for our results on meningitis. Concerning hypotonia, no evidence that proves a direct link between lack of breastfeeding and low muscle neonatal tone but scattered data from different sources show that many neural outcomes are facilitated by HM feeding (12). On the one side, it has been reported that a high proportion of floppy infants also show shallow measurements of muscle strength (13, 14), and systematic reviews confirm the positive association between breastfeeding and body strength or balance beyond infancy (15, 16). In particular, in the long-term, adolescents who were breastfed build stronger muscles than those who were not breastfed (17). In the medium-term or short-term, there is substantial evidence of the positive impact of breastfeeding on masseter muscle function among toddlers or infants (18, 19). The combination of the negative correlation between hypotonia and muscle strength and the positive correlation between the history of breastfeeding and subsequent muscle strength may suggest some degree of correlation between lack of HM feeding and neonatal hypotonia.

Regarding our findings on neutrophil counts, we can refer to the well-known link between reactive oxygen species and high neutrophil-to-lymphocyte ratios. This association has been seen in critically ill patients, which is accompanied by a cascade of events that leads to tissue damage and increases the severity of the condition (20). In this field of study, we report no differences between the two groups of infants for the rest of the leukocytes.

While low arterial blood pressure of neonates with sepsis is linked to an increased risk of adverse outcomes (21), treating neonatal hypotension using vasopressors remains controversial (22–24). Hence, there is a consensus to promote a thorough examination of the pathophysiological mechanisms underlying low systemic perfusion to help clinicians decide which treatment strategy to use (25).

Preterm infants have myocardial dysfunction as well as immature vasculature (26). We are still learning HM feeding can have cardioprotective effects on sick neonates. The mechanism by which HM may reverse those cardiovascular changes is one of the crucial elements that is still absent. Our study may add to robust data on the benefits of HM exposure for the cardiovascular morphology of the high-risk newborn and is also consistent with findings on oxidative stress-related diseases in newborns.

The link between HM feeds and enhanced brain or lung development among high-risk neonates (27, 28) has prompted a renewed interest in optimal feeding to improve the cardiovascular health of such neonates. Preterm infants with higher exposure to HM show enhanced cardiovascular performance by age one year (29) and a more favorable cardiac structure during adolescence and adulthood (30, 31). Our study provides further evidence of the beneficial association between early HM feeding and cardiovascular function across the neonatal developmental stage of sick neonates, which is characterized by systolic and diastolic dysfunction coupled with maladaptive vasculature (32, 33).

The massive release of pro-inflammatory molecules noted among newborn infants with bloodstream infection leads to systemic inflammatory reaction (34) in organs distant from the initial insult, involves the endothelial cells, and affects the vascular tone (3, 4, 35). Ideally, this inflammatory response is followed by a compensatory anti-oxidative response, which represents the biological attempt to prevent inflammation from becoming the dominant reaction and leading to multiorgan dysfunction (5, 36).

An increased oxidized to reduced glutathione (GSSG/GSH) ratio in blood is considered a sign of oxidative stress (37). GSSG/GSH ratios are significantly higher in each phase of septic shock in infants or children; they spike during the overspill inflammatory state and then level off (38). Accordingly, significantly higher levels of GSSG are found among critically ill infants than among matched controls. On the other hand, premature birth removes the fetus from the protected, hypoxic intrauterine milieu. It exposes the sick preterm infant to free radical injury from using high-inspired oxygen fractions or inhaled nitric oxide or from reactive oxidative species produced by septic shock (39).

Furthermore, the lack of transfer of antioxidants across the placenta during the last stages of pregnancy, coupled with their weakened capacity to synthesize antioxidant defenses, renders such infants more vulnerable to oxidative stress. As expected, it has been confirmed that preterm newborns have lower plasma GSH than full-term newborns (40). Thus, the only compensatory mechanism for the sick neonate to cope with these compromised or incomplete antioxidant systems is the free radicals scavenging ability provided by HM (41).

Studies on adults over the past two decades report that the introduction of enteral nutrition, even if the patient requires continued inotropic support, improves both cardiac index and splanchnic blood flow while restores the gastrointestinal dysfunction and reduces hospital mortality rate, which is more visible in patients treated with multiple inotropes (42–44). In respect of Pediatric Intensive Care Units, when early enteral feeding was started in critically ill children with heterogeneous diagnoses, retrospective investigations showed a decreased length of stay in the unit, decreased mortality, and reduced use of inotropes (45, 46). Consistently, the consensus is that inotropes are not a contraindication in carefully monitored early enteral feeding of unstable adults (47). Recent pediatric sepsis guidelines encourage the start of early enteral nutrition based on the child's clinical situation, given that the scant literature on the subject suggests that it is both safe and beneficial (48).

Attention should be drawn to the lack of clinical trials on early enteral feeding of neonates on inotropes. To our knowledge, only a recent randomized trial has addressed this topic. Rao et al. (49) conducted a study on 200 late preterm infants on inotropes randomly assigned to an enteral feed study group or an intravenous (IV) fluid group (one hundred each). After the infant had stable circulation for 6 h, the enteral feed group began feeding with expressed breast milk. The feeding guidelines were individualized based on the infant's clinical status, and there was no increase in the initial volume for at least 24 h. When babies were fed 80 ml/kg/day, feeding intervals of 4 h were replaced with 2 h intervals. From 100 to 160 ml/kg/day, feed volumes were advanced at less than 30 ml/kg/day. Neonates in the IV fluid group were not given any feed. All neonates in enteral feed group were expressed breast milk-fed and were closely monitored until death or discharge. Mortality in the IV fluid group was significantly higher than in the enteral group (30.4% vs. 11.7%, respectively; *p* = 0.002), and the mean duration of hospital stay was longer in the IV fluid group (15.5 days vs. 13.4 days in the enteral feed group; *p* = 0.013). As a result, the authors conclude that neonates can tolerate enteral feed up to dopamine 10 mcg/kg/min and dobutamine 7.5 mcg/kg/min. The practical advice is that enteral feed can be safely administered to neonates on inotropes.

According to currently limited evidence on unstable neonates, some neonatal guidelines favor withholding feeds for babies on inotropes (50), while others prefer to continue enteral feeds even if inotropic support occurs (51). Concerning clinical practice, a survey of 60 Spanish NICUs unveils that 100% delay enteral feeding when there is a hemodynamic failure (52). Considering that we analyzed data from a purely observational cohort, our findings do not allow at this stage to suggest different approaches to minimize inotrope treatments with uncertain cognitive outcomes (53). However, this study contributes evidence on the potential role of very early HM feeding in improving the quality of care of newborn infants with signs of shock and might boost further research on the most effective feeding practices for this group of neonates.

Our study has several limitations. First, we acknowledge that data collection on HM needs to be more specific regarding the vast variability of what this could constitute over the first weeks of life. However, the association between breastfeeding and short- or long-term health outcomes has received considerable scrutiny. A number of studies have suggested that ever vs. never HM feeding may be associated with a decreased risk of mother's carcinoma (54), cardiovascular health in schoolchildren (55), or retinopathy of prematurity among neonates (56), even after adjustment for potential confounders. Second, our study was limited because we did not include feeding duration in our definition of HM feeding. From a methods perspective, we had no information on the feeds in temporal relation to the treatment decision. Previous studies have shown that the rate of breastfeeding initiation may be the same for infants admitted to the well-newborn nursery and the NICU and also that early cessation of breastfeeding is due to the newborn's sickness in less than 5% of cases (57). Hence, other authors have pointed to links between feeding type and neonatal conditions that are rare during the first week of life, based on data similar to ours, despite the lack of detailed information on the periods in which their patients were fed HM (56).

Third, we also had no information on the number of doses and the timeline of any vasopressor treatment. Moreover, the questionnaire did not collect the name of the vasopressor used. The reasons for prescribing vasopressors were also missing, although probably was due to hypotension. Fourth, the study reflects practices from a long time, which might have changed a decade later. Fifth, as this is a descriptive study, residual confounding may not have been identified. Sixth, we did not account for social characteristics that could impact the outcomes. Seventh, if further clinical, laboratory, or ultrasound data could be examined, our results would have been conclusive, but we must admit that hypotension was the only sign of compromised cardiovascular function that was collected.

## Conclusion

Our results show that any HM feeding is associated with a decrease in the need for vasoactive medications in sepsis-affected newborns. This observation suggests that the antioxidant system that is part of HM may protect against inflammatory pathways that impact the vascular tone in sepsis and encourages further research to determine whether HM feeds directly affect neonatal cardiovascular maturation patterns.

## Data Availability

The raw data supporting the conclusions of this article will be made available by the authors, without undue reservation.
